# miR-146a deficiency does not aggravate muscular dystrophy in *mdx* mice

**DOI:** 10.1186/s13395-019-0207-0

**Published:** 2019-08-14

**Authors:** Iwona Bronisz-Budzyńska, Katarzyna Chwalenia, Olga Mucha, Paulina Podkalicka, Karolina-Bukowska-Strakova, Alicja Józkowicz, Agnieszka Łoboda, Magdalena Kozakowska, Józef Dulak

**Affiliations:** 1 Department of Medical Biotechnology, Faculty of Biochemistry, Biophysics and Biotechnology, Jagiellonian University, Gronostajowa 7, 30-387 Krakow, Poland; 2 Department of Clinical Immunology and Transplantology, Institute of Paediatrics, Medical College, Jagiellonian University, Wielicka 265, 30-663 Krakow, Poland

**Keywords:** miR-146a, Skeletal muscle, *mdx*, Duchenne muscular dystrophy, Inflammation, Regeneration

## Abstract

Duchenne muscular dystrophy (DMD) is a genetic disease evoked by a mutation in the dystrophin gene. It is associated with progressive muscle degeneration and increased inflammation. Up to this date, mainly anti-inflammatory treatment is available for patients suffering from DMD. miR-146a is known to diminish inflammation and fibrosis in different tissues by downregulating the expression of proinflammatory cytokines. However, its role in DMD has not been studied so far.

In our work, we have generated mice globally lacking both dystrophin and miR-146a (miR-146a^−/−^*mdx*) and examined them together with wild-type, single miR-146a knockout and dystrophic (*mdx*—lacking dystrophin) mice in a variety of aspects associated with DMD pathophysiology (muscle degeneration, inflammatory reaction, muscle satellite cells, muscle regeneration, and fibrosis).

We have shown that miR-146a level is increased in dystrophic muscles in comparison to wild-type mice. Its deficiency augments the expression of proinflammatory cytokines (IL-1β, CCL2, TNFα). However, muscle degeneration was not significantly worsened in *mdx* mice lacking miR-146a up to 24 weeks of age, although some aggravation of muscle damage and inflammation was evident in 12-week-old animals, though no effect of miR-146a deficiency was visible on quantity, proliferation, and in vitro differentiation of muscle satellite cells isolated from miR-146a^−/−^*mdx* mice vs. *mdx*. Similarly, muscle regeneration and collagen deposition were not changed by miR-146a deficiency. Nevertheless, the lack of miR-146a is associated with decreased *Vegfa* and increased *Tgfb1*.

Overall, the lack of miR-146a did not aggravate significantly the dystrophic conditions in *mdx* mice, but its effect on DMD in more severe conditions warrants further investigation.

**Electronic supplementary material:**

The online version of this article (10.1186/s13395-019-0207-0) contains supplementary material, which is available to authorized users.

## Background

Duchenne muscular dystrophy (DMD) is an X chromosome-associated monogenic disease, caused by mutations in a gene encoding dystrophin, leading to the lack of functional protein [1, 2]. Dystrophin is the major intracellular part of the dystrophin-glycoprotein complex, which links extracellular matrix through sarcolemma to multiple cytoskeletal proteins, ensuring signal transduction and mechanical stability of myofibres during contraction [3–5].

Although in healthy skeletal muscle dystrophin constitutes only 0.002% of total protein mass [6], its deficiency causes detrimental effects. The damage of sarcolemma followed by the degeneration of muscle fibres are the primary results of the lack of dystrophin [5, 7]. Injuries occur especially during contraction, due to the changes in the localisation of membrane proteins which lead to the increased mechanical vulnerability and permeability of the sarcolemma [7, 8]. As a result, degenerating myofibres accumulate immunoglobulins IgG and IgM [8], whereas during the necrosis, they release proteins (e.g. lactate dehydrogenase (LDH) and creatine kinase (CK)) that can be found afterwards in the plasma [1, 9, 10]. Consequently, massive inflammation and leukocyte infiltration of the tissue take place [5, 7, 11], amplifying sarcolemma damage of dystrophic myofibres [12]. Neutrophils and phagocytic macrophages of pro-inflammatory M1 phenotype start to invade dystrophic skeletal muscle, subsequently accompanied by pro-regenerative and anti-inflammatory M2 subpopulation [12, 13]. Persistent membrane instability and proinflammatory cytokines induce the expression of major histocompatibility complex (MHC I and II) on muscle cells, and afterwards recruitment of T_h_ and T_c_ lymphocytes, that further contribute to muscle damage [11, 14] also by secretion of tumour necrosis factor-α (TNFα) and interferon-γ (IFNγ) cytokines that induce proinflammatory phenotype in macrophages [13–15]. T_reg_ lymphocytes are also elevated in dystrophic muscles; however, by secretion of immunosuppressive IL-10 and reduction of IFNγ expression by T_h_ lymphocytes, they play there an anti-inflammatory role [11, 16].

In response to the repetitive primary and secondary damage of muscle tissue, the process of muscle regeneration is induced [5, 7]. It is strictly dependent on the muscle satellite cells (SCs)—progenitors of skeletal muscle tissue that became activated upon injury and give rise to myoblasts [17, 18]. The muscle recovery is controlled by a group of muscle regulatory transcription factors (MRFs, including among them myoblast determination protein 1–MyoD and myogenin) and muscle-specific microRNAs (miRNAs, so-called myomirs, miR-1, miR-133a/b, and miR-206) [17, 18]. Myoblasts differentiate, fuse to each other, and develop into myofibres, upregulating the expression of proteins characteristic for regenerating (e.g. embryonic myosin, eMHC/Myh3) and mature (e.g. myosin heavy chain, MyHC) myofibres [17–19]. Until recently, dystrophin was thought to be one of these proteins, expressed only in myotubes and myofibres, but its presence was, in fact, confirmed already in SCs [20]. Its lack in SCs of dystrophic muscles results in the impaired polarity of SCs, loss of asymmetric division, reduced generation of myogenic progenitors, and finally impaired muscle regeneration [20].

Abnormal regeneration which cannot effectively compensate chronic muscle degeneration, together with the persistent inflammatory infiltration, lead in dystrophic muscles to excessive deposition of extracellular matrix (ECM) proteins, in the process called fibrosis [21, 22]. When properly controlled, it is necessary to provide a scaffold for the correct structure of newly formed muscle tissue and to ensure proper transmembrane signalling [21, 22]. However, during dystrophy progression, fibroblasts and myofibroblasts, generated from fibro-adipogenic progenitors (FAPs), produce high levels of proteins like collagens and fibronectin in response to elevated transforming growth factor-β (TGF-β) expression [21, 23]. Abnormal accumulation of connective tissue within skeletal muscles perturbs the microenvironment of the injured tissue, diminishes the access to nutrients, and limits the availability of target muscle cells for the treatment [21].

Multiple rounds of degeneration-regeneration events occurring with increasing age, accompanied by elevated inflammatory reaction and fibrosis lead ultimately to the poor repair response and the loss of muscle function [7, 11, 21]. This, in turn, results in premature death, often due to respiratory or cardiac failure [24]. Since the current search for an ultimate treatment for the disease is unsuccessful, reduction of deleterious secondary effects, leading to improvement of lifespan and life quality, are the main field of research [7, 24, 25].

We have recently shown that one of microRNAs, namely miR-146a, is constantly upregulated in *mdx* mice—a murine model of DMD [9]. Research done in different tissues show that miR-146a negatively regulates inflammation, by inhibiting activators of NF-κB pathway—interleukin-1 receptor-associated kinase 1 (IRAK1) and TNF receptor-associated factor 6 (TRAF6) [26–29]. In this manner, miR-146a leads to the decreased production of proinflammatory cytokines [30–34] and affects macrophage-dependent inflammatory response [30, 35], as well as activity of NK cells [33, 34] and T cells [29, 36–38]. Moreover, miR-146a was proved to inhibit skeletal [39] and cardiac [40] muscle fibrosis acting as a negative regulator of TGF-β signalling pathway [39]. Finally, miR-146a is upregulated in murine myoblasts which present decreased differentiation due to heme oxygenase-1 overexpression [41]. In the same cell line, miR-146a was shown to intensify proliferation and reduce differentiation by affecting Numb [42], an inhibitor of a Notch signalling pathway, which regulates postnatal myogenesis [43, 44].

Despite these known properties of miR-146a, suggesting it as a potential target of anti-dystrophic therapies, its role in muscular dystrophy has not been addressed so far. In the current study, we have therefore investigated what is the effect of global miR-146a deficiency in *mdx* mice.

## Methods

### Animal models

All animal procedures and experiments were performed in accordance with national and European legislation, after approval by the 1st Local Ethical Committee on Animal Testing (approval number: 66/2013). Animals were kept in specific-pathogen-free standard conditions with water and food available ad libitum.

*Mdx* mice C57BL/10ScSn-*Dmd*^*mdx*^/J and control mice C57BL/10ScSnJ (WT), as well as miR-146^−/−^ B6(FVB)-*Mir146*^*tm1.1Bal*^/J mice, were purchased from the Jackson Laboratory. To generate miR-146a^−/*−*^*mdx* (mice deficient for both miR-146a and dystrophin), homozygous miR-146a^−/*−*^ male mice were bred to homozygous *Dmd*^*mdx/mdx*^ female mice, to generate miR-146a^+/*−*^Dmd^*mdx*/+^ female mice or miR-146a^+/*−*^Dmd^*mdx*/Y^ male mice, which were bred together to obtain miR-146a^−/*−*^*mdx* mice at mixed background C57BL/10ScSn and B6(FVB) (F3). As controls, miR-146a^+/+^Dmd^+/Y^ (WT), miR-146a^+/+^Dmd^mdx/Y^ (*mdx*), miR-146a^−/−^Dmd^+/Y^ (miR-146a^−/−^) at mixed background were used (F3 generation). The crossing of mice to generate double knockouts was hence done accordingly to other studies in which *mdx* mice were crossed with relevant knockouts [9, 45–50]. 10- to 12-week-old male littermates or age-matched mice were used for the analysis. For experiment analysing the effect of miR-146a deficiency in older animals, 24-week-old mice were used. Genotyping of animals was performed by PCR on the DNA isolated from the tails.

### Histological analysis

*Gastrocnemius muscles* (GM) were placed in 10% formalin for 48 h or preserved in OCT freezing medium, in isopentane cooled in a bath of liquid nitrogen. Four-micrometre-thick sections or 10-μm-thick sections were cut from each paraffin-embedded tissue and frozen muscles, respectively, with the muscle fibres oriented in a transverse direction. Muscle sections were subjected to haematoxylin and eosin (HE) or Masson’s trichrome staining, accordingly to published protocols [51]. Inflammation, regeneration, and fibrosis were based on the previously described arbitrary scale [51].

### Plasma CK and LDH measurement

Plasma was obtained from the blood collected from the *vena cava* just before terminal procedure and muscles harvesting. The activity of CK and LDH was measured using diagnostic Liquick Cor-CK and Liquick Cor-LDH kit, respectively (P.Z. CORMAY), as previously described [9, 51].

### Immunohistofluorescent (IHF) stainings

GM was snap-frozen in tissue freezing compound (OCT) in pre-chilled isopentane bath cooled with liquid nitrogen. Frozen tissues were cryosectioned (10 μm) using cryostat (Leica).

Necrotic fibres (accumulating IgG and IgM) or regenerating fibres (positive for embryonic myosin chain, eMHC) were stained on cryosections. Muscle frozen sections were blocked with 10% goat serum (Sigma-Aldrich), 5% bovine serum albumin (BioShop), and with M.O.M.™ (Mouse On Mouse Ig blocking reagent, Vector Laboratories) for 1 h at room temperature. Afterwards, sections were incubated with rat anti-mouse laminin 2α primary antibody (1:500; 4H8-2, Abcam), mouse anti-mouse eMHC primary antibody (1:100, F1.562, DSHB) for 1 h at 37 °C, followed by three washes with PBS (5 min each) and 1-h-incubation with goat anti-rat AlexaFluor568 (1:1000, A-11077, Thermo Fisher Scientific), goat anti-mouse AlexaFluor488 (1:500, A11008, Thermo Fisher Scientific), and goat anti-mouse IgG/IgM/IgA-AlexaFluor488 (1:50, A-10667, Thermo Fisher Scientific). Finally, sections were washed with PBS, counterstained with Hoechst 33258 (10 μg/ml, Sigma-Aldrich), and covered with fluorescence mounting medium (Dako). The percentage of necrotic fibres or regenerating fibres was assessed among the total myofibre number.

Dystrophin expression was checked on frozen cryosections fixed by ice-cold acetone. Sections were blocked with 10% goat serum and 3% bovine serum albumin for 1 h; primary rabbit anti-mouse dystrophin (1:100; ab15277, Abcam) was applied overnight followed by three washes with PBS and 1-h-incubation with donkey anti-rabbit AlexaFluor488 (1:500, A21206, Thermo Fisher Scientific). Finally, sections were washed with PBS, counterstained with Hoechst 33258 (10 μg/ml), and covered with fluorescence mounting medium.

For Pax7, staining sections were fixed for 20 min in 4% paraformaldehyde (Santa Cruz) and followed the one wash with PBS and fixed and permeabilised with cold methanol (POCH S.A.) for 6 min at − 20 °C. Then, after two washes with PBS, retrieval of antigens was performed in the citric buffer. After two washes with PBS, samples were blocked in 2.5% bovine serum albumin for 30 min and M.O.M.™ for the next 30 min. After two washes with PBS, primary antibodies against Pax7 (1:100, Pax7-c, DSHB) and laminin 2α (1:1000, L9393, Sigma-Aldrich) were applied overnight at 4 °C in 0.1% BSA. After two washes with PBS (5 min each), the sections were incubated with secondary goat anti-mouse AlexaFluor488 (1:500, A11008, Thermo Fisher Scientific) and goat anti-rabbit AlexaFluor568 (1:500, A-11077, Thermo Fisher Scientific) for 30 min at room temperature in 0.1% BSA antibodies. Finally, sections were washed with PBS, counterstained with Hoechst 33258 (10 μg/ml), and covered with fluorescence mounting medium. The ratio of Pax7^+^ cells/myofibre was assessed among the total myofibre number, and at least 8 fields of view were analysed.

### Analysis of mononucleated cells populations in skeletal muscles by flow cytometry

Cells for flow cytometry were prepared as previously described [9, 51]. Briefly, hind limb muscles were pooled, minced, and digested with 5 mg/ml Collagenase IV (Gibco; Invitrogen) and 1.2 U/ml Dispase (Gibco; Invitrogen) at 37 °C. The cell suspension was filtered through a 100-μm cell strainer, and cells were pelleted after centrifugation.

For cytometric analysis of SCs, pelleted cells after skeletal muscle digestion were resuspended in PBS + 2% fetal bovine serum (FBS) and then incubated for 30 min on ice with rat anti-mouse α7integrin-PE (1:15, 334,908, R&D Systems), rat anti-mouse CD34-AlexaFluor700 (1:30, RAM34, eBioscience), rat anti-mouse CD45-APC-eFluor780 (1:30, 30-F11, eBioscience), rat anti-mouse CD31-PE (1:30, MEC13.3, BD Biosciences), and rat anti-mouse Sca-1-PE-Cy7 (1:30, D7, eBioscience) to assess CD45^−^CD31^−^Sca1^−^α7integrin^+^CD34^+^ and CD45^−^CD31^−^Sca1^−^α7integrin^+^CD34^−^ SCs [9, 51]. For intracellular protein detection, cell fixation and permeabilisation was done with BD IntraSure™ Kit (BD Biosciences) according to the vendor’s protocol. Primary rabbit polyclonal anti-mouse Numb (1:200, C29G11, Cell Signalling) and appropriate goat anti-rabbit AlexaFluor488 secondary antibody (1:400, A11008, Thermo Fisher Scientific) were used. A negative control without primary antibody was prepared. Cell cycle phases were determined based on Hoechst 33342 staining (10 μg/ml). The stained cells were analysed using Fortessa flow cytometer (BD Biosciences), with FACSDiva (BD Biosciences).

For cytometric analysis of macrophage, monocyte, and granulocyte populations, pelleted cells after skeletal muscle digestion were resuspended in PBS + 2% FBS and then incubated with the following antibodies for 30 min on ice: rat anti-mouse CD45-APC-eFluor780 (1:30, 30-F11, eBioscience), rat anti-mouse F4/80-APC (1:30, BM8, eBioscience), rat anti-mouse MHCII-PE-Cy7 (1:30, M5/114.15.2, BD Bioscience), rat anti-mouse 11b-PE (1:30, M1/70, eBioscience), rat anti-mouse CD206-PerCP/Cy5.5 (1:30, C0682C2, BioLegend), rat anti-mouse Ly6C-AlexaFluor488 (1:30, HK1.4, BD Biosciences), and rat anti-mouse Ly6G-PE (1:30, 1A8, BioLegend). Cells were fixed with BD IntraSure™ Kit.

For cytometric analysis of NK and lymphocyte populations, cells pelleted after skeletal muscle digestion were resuspended in PBS + 2% FBS and then incubated with the following antibodies for 30 min on ice: rat anti-mouse CD45-APC-eFluor780 (1:30, 30-F11, eBioscience), hamster anti-mouse CD3e-PE-Cy7 (1:30, 145-2C11, eBioscience), mouse anti-mouse NK1.1-FITC (1:30, PK136, BioLegend), rat anti-mouse CD4-PerCP-Cyanine 5.5 (1:30, RM4-5, BD Biosciences), rat anti-mouse CD8a-AlexaFluor700 (1:30, 53-6.7, BioLegend), and rat anti-mouse CD25-PE (1:30, PC61, BD Biosciences). After fixation and permeabilisation, rat anti-mouse FoxP3-APC (1:30; FJK-16 s, eBioscience) was applied.

Before the flow cytometry analysis, all cells were additionally stained with Hoechst 33342 (10 μg/ml).

### Isolation of SCs by fluorescence-activated cell sorting (FACS)

For isolation of SCs by FACS sorting, skeletal muscles from hind limbs were prepared similarly as for flow cytometry analysis, resuspended in PBS + 2% FBS, and then incubated with the following antibodies for 30 min on ice: rat anti-mouse α7integrin-APC (1:15, 334,908, R&D Systems), rat anti-mouse CD34-FITC (1:30, RAM34, eBioscience), rat anti-mouse CD45-PE (1:30, 30-F11, BD Biosciences), rat anti-mouse CD31-PE (1:30, MEC13.3, BD Biosciences), and rat anti-mouse Sca-1-PE-Cy7 (1:30, D7, eBioscience). After incubation, cells were washed, filtered through a 40-μm cell strainer, and resuspended in PBS + 2% FBS with Hoechst 33342 (10 μg/ml) and 7-AAD (1:40, BD Biosciences). Cells were sorted with MoFlo XDP (Beckman Coulter) cell sorter.

### SCs cell culture, proliferation, and differentiation

Cell culture, analysis of in vitro proliferation by 5-ethynyl-2′-deoxyuridine incorporation (EdU, 5-ethynyl-2′-deoxyuridine, Thermo Fisher Scientific), in vitro differentiation, and immunocytochemical fluorescent staining (ICC-F) for myosin-heavy chain (MyHC) were performed as previously described [9, 51]. The fusion index was defined as a percentage of nuclei within myotubes (≥ 3 nuclei) related to the total number of nuclei.

### Total RNA isolation and qRT-PCR

Total RNA isolation from GM and qRT-PCR for both mRNAs and miRNAs were performed as previously described [9, 51]. The primers recognising mouse *I1b* (5′- CCGACAGCACGAGGCTTT-3′; 5′- CTGGTGTGTGACGTTCCCATT-3′), *Ccl2* (5′-CCCAATGAGTAGGCTGGAGA-3′; 5′-TCTGGACCCATTCCTTCTTG-3′), *Tnf* (5′-ACGTCGTAGCAAACCACC-3′; 5′-TAGCAAATCGGCTGACGGT-3′), *Myod1* (5′-GCTGCCTTCTACGCACCTG-3′; 5′-GCCGCTGTAATCCATCATGC-3′), *Myog* (5′-CAGTACATTGAGCGCCTACAG-3′; 5′-GGACCGAACTCCAGTGCAT-3′), *Myh3* (5′- TCTAGCCGGATGGTGGTCC-3′; 5′-GATTGTAGGAGCCACGAAA-3′), *Col1a1* (5′-CGATCCAGTACTCTCCGCTCTTCC-3′; 5′-ACTACCGGGCCGATGATGCTAACG-3′), *Tgfb1* (5′-CGCAACAACGCCATCTATGAG-3′; 5′- TTCCGTCTCCTTGGTTCAGC-3′), *Vegfa* (5′-ATGCGGATCAAACCTCACCAA-3′; 5′-TTAACTCAAGCTGCCTCGCCT-3′), *Mmp9* (5′-TGTGGATGTTTTTGATGCTATT-3′; 5′-CGGAGTCCAGCGTTGCA-3′), and *Eef2* for normalisation (elongation factor 2) (5′-AGAACATATTATTGCTGGCG-3′; 5′-CAACAGGGTCAGATTTCTTG-3′) were used. Forward primers recognising muscle-specific murine miRNAs miR-206 (5′-TGGAATGTAAGGAAGTGTGTGG-3′), miR-146a (5′-CGTGAGAACTGAATTCCATGGGTT-3′), miR-133a (5′-TTGGTCCCCTTCAACCAGCTGT-3′), and miR-1 (5′-GCTGGAATGTAAAGAAG TATGTAT-3′) were used. Universal reverse primer for miRNAs’ quantitative RT-PCR was supplied by a vendor. Gene expression was normalised to a constitutive small RNA U6 (5′-CGCAAGGATGACACGCAAATTC-3′).

### Protein analysis

To assess vascular endothelial growth factor A (VEGF) protein level in gastrocnemius lysate, the Luminex™ platform was used. VEGF was measured according to the manufacturer’s instructions (Life Technologies) and the results were calculated as pg/mg of total protein.

### Statistics

Data are presented as mean ± SEM. Differences between groups were tested for statistical significance using the unpaired two-tailed Student’s *t* test. *p* ≤ 0.05 was considered significant. Grubb’s test was used to identify significant outliers.

## Results

### miR-146a is elevated in dystrophic muscles and its lack increases expression of proinflammatory genes

**Fig. 1:**
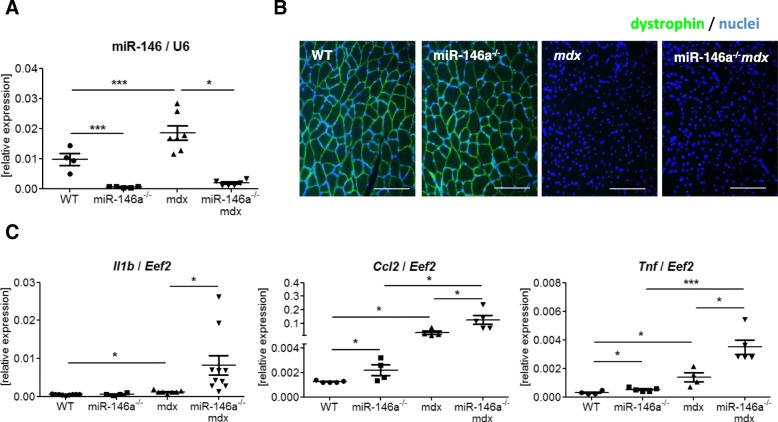
General phenotype of muscle of WT, miR-146a^−/−^, *mdx*, and miR-146a^−/−^*mdx* mice. **a** Level of miR-146a in GM; qRT-PCR. **b** Dystrophin expression in GM; IHF staining; representative photos; *n* = 3–5. **c** *Il1b*, *Ccl2*, *Tnf* level in GM; qRT-PCR. Mean +/− SEM; *n* = 3–11; *− *p* ≤ 0.05; **− *p* ≤ 0.01; ***− *p* ≤ 0.001. Scale bars 100 μm

To confirm the miR-146a deficiency of miR-146a^*−/−*^*mdx* mice generated in our lab, qRT-PCR was performed (Fig. 1a). miR-146a^*−/−*^ and miR-146a^*−/−*^*mdx* animals lack the expression of miR-146a (Fig. 1a). Similarly, their dystrophic phenotype was verified and both *mdx* and miR-146a^*−/−*^*mdx* did not express dystrophin protein (Fig. 1b). Additionally, we have analysed the expression of genes that were reported previously to be affected on mRNA level by miR-146a [30, 31, 33, 52]. Accordingly, increased mRNA level of proinflammatory cytokines such as *Il1b*, *Ccl2,* and *Tnf* was found in miR-146a-deficient muscles (Fig. 1c).

### miR-146a deficiency does not significantly aggravate muscle degeneration and inflammatory reaction in dystrophic muscles

**Fig. 2:**
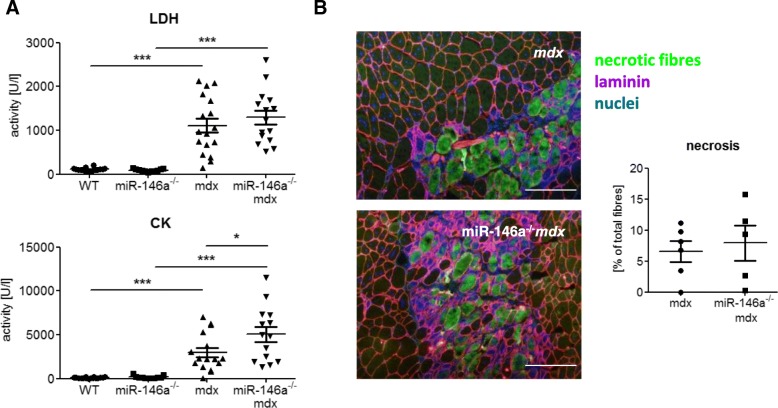
Muscle degeneration of WT, miR-146a^−/−^, *mdx*, and miR-146a^−/−^*mdx* mice. **a** Activity of LDH and CK in plasma; activity test. **b** Necrosis in GM; IHF staining of IgM and IgG binding and its calculation. Mean +/− SEM; *n* = 5–18; *− *p* ≤ 0.05; **− *p* ≤ 0.01; ***− *p* ≤ 0.001; scale bars 100 μm

Degeneration of skeletal muscle was measured basing on markers released to blood (Fig. 2a) and determination of the percentage of necrotic fibres (Fig. 2b). No statistically significant differences were detected in LDH activity, as well as in the level of necrosis in GM of dystrophic mice lacking additionally miR-146a in comparison to *mdx* animals (Fig. 2a, b). However, stronger muscle damage can be noted in dystrophic muscles in the absence of miR-146a, as evidenced by an increase in CK (Fig. 2a).

**Fig. 3:**
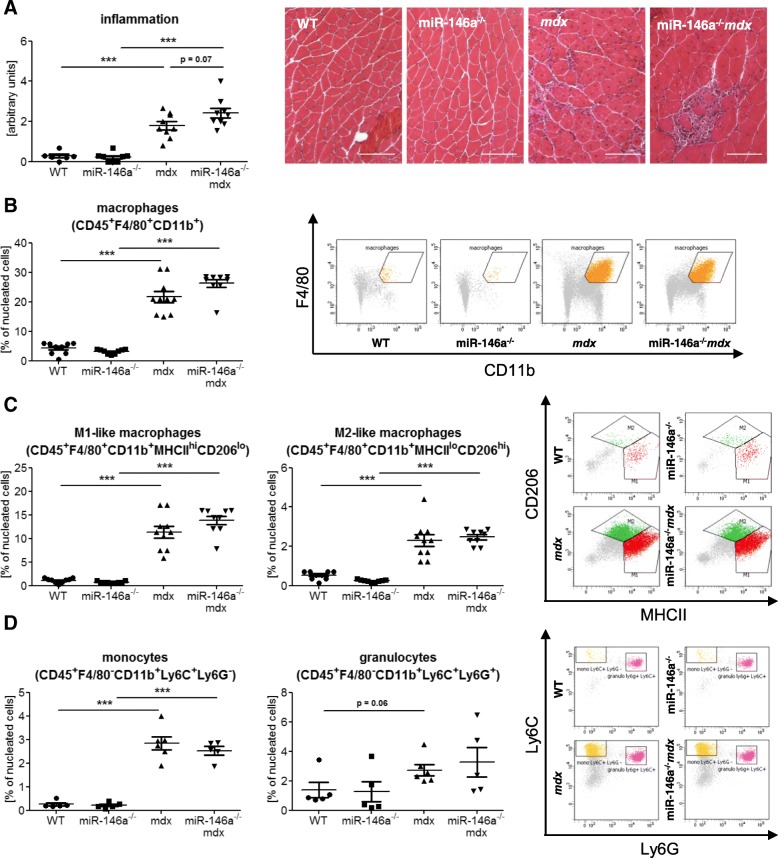
Infiltration of WT, miR-146a^−/−^, *mdx*, and miR-146a^−/−^*mdx* hind limb muscle with leukocytes, macrophages, monocytes, and granulocytes. **a** Semi-quantitative analysis of inflammation in GM muscle; HE staining; representative photos. **b** Percentage of CD45^+^F4/80^+^CD11b^+^ macrophages; flow cytometry. **c** Percentage of M1-like macrophages (CD45^+^F4/80^+^CD11b^+^MHCII^hi^CD206^lo^) and M2-like macrophages (CD45^+^F4/80^+^CD11b^+^MHCII^lo^CD206^hi^); flow cytometry. **d** Percentage of monocytes (CD45^+^F4/80^−^CD11b^+^Ly6C^+^Ly6G^−^) and granulocytes (CD45^+^F4/80^−^CD11b^+^Ly6C^+^Ly6G^−^); flow cytometry. Mean +/− SEM; *n* = 4–10; *− *p* ≤ 0.05; ***− *p* ≤ 0.001. Scale bars 100 μm

**Fig. 4:**
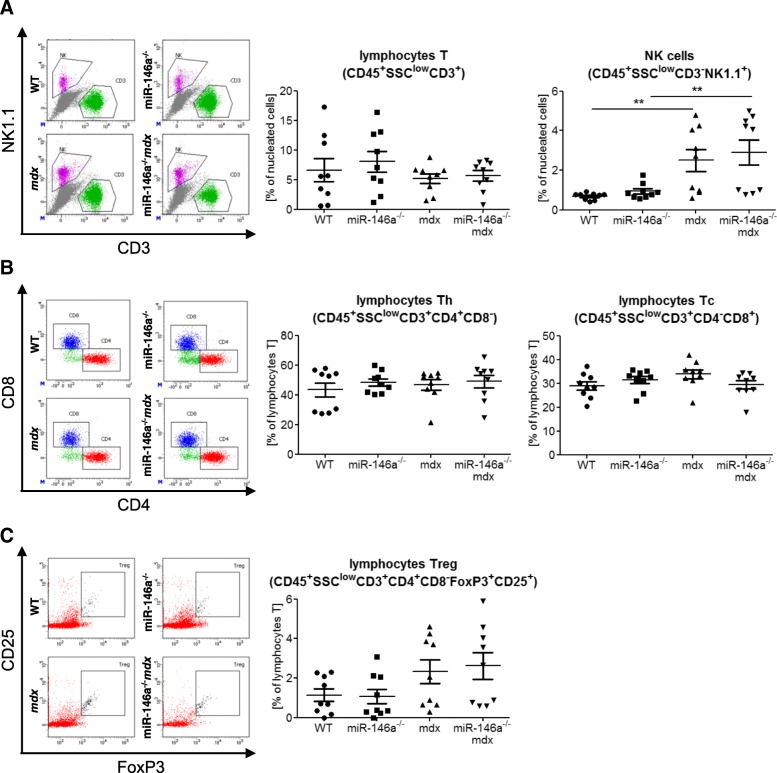
Infiltration of WT, miR-146a^−/−^, *mdx*, and miR-146a^−/−^*mdx* hind limb muscles with lymphocytes and NK cells. **a** Percentage of lymphocytes T (CD45^+^SSC^lo^CD3^+^NK1.1^−^) and NK cells (CD45^+^SSC^lo^CD3^−^NK1.1^+^); flow cytometry. **b** Percentage of lymphocytes T_h_ (CD45^+^SSC^lo^CD3^+^CD4^+^CD8^−^) and T_c_ (CD45^+^SSC^lo^CD3^+^CD4^−^CD8^+^); flow cytometry. **c** Percentage of lymphocytes T_reg_ (CD45^+^SSC^lo^CD3^+^CD4^+^CD8^−^Foxp3^+^CD25^+^); flow cytometry. Mean +/− SEM; *n* = 9; **− *p* ≤ 0.01

To assess the level of inflammatory reaction occurring in skeletal muscle of WT, miR-146a^*−/−*^, *mdx*, miR-146a^*−/−*^*mdx* animals, the histological analysis was performed (Fig. 3a). Since in miR-146a^*−/−*^*mdx* mice a tendency toward stronger muscle degeneration and inflammatory infiltration was shown (Fig. 3a), as well as raised expression of genes associated to inflammatory reaction was observed (Fig. 1c), we decided to analyse leukocyte populations of cells within the skeletal muscles of hind limbs of mice of 4 genotypes (Figs. 3 and 4).

The percentage of macrophages (CD45^+^F4/80^+^CD11b^+^), the cells that mainly infiltrate injured muscle, was increased in *mdx* and miR-146a^*−/−*^*mdx* in comparison to WT and miR-146a^*−/−*^, respectively (Fig. 3b). However, no additional differences were shown in *mdx* mice additionally lacking miR-146a in comparison to dystrophic animals (Fig. 3b), although again the borderline increase in inflammation score is visible in the absence of miR-146a (Fig. 3a). M1-like macrophages (CD45^+^F4/80^+^CD11b^+^MHCII^hi^CD206^lo^) and M2-like macrophages (CD45^+^F4/80^+^CD11b^+^MHCII^lo^CD206^hi^) were also investigated (Fig. 3c). Although a strong increase of these cells in dystrophic mice was evident, no further changes were detected in miR-146a^*−/−*^*mdx* compared to *mdx* (Fig. 3c). Similar alterations were shown in monocytes (CD45^+^F4/80^−^CD11b^+^Ly6C^+^Ly6G^−^) found within skeletal muscles, whereas no significant differences were visible between four genotypes in case of granulocytes (CD45^+^F4/80^−^CD11b^+^Ly6C^+^Ly6G^+^) infiltrating skeletal muscles (Fig. 3d); however, the clear tendency for granulocytes increase is noted in muscles lacking dystrophin (Fig. 3d).

The number of NK cells (CD45^+^SSC^low^CD3^−^NK1.1^+^) was increased in *mdx* and miR-146a^*−/−*^*mdx* in comparison to WT and miR-146a^*−/−*^, respectively (Fig. 4a). T (CD45^+^SSC^low^CD3^+^), T_h_ (CD45^+^SSC^low^CD3^+^CD8^−^CD4^+^), and Tc (CD45^+^SSC^low^CD3^+^CD8^+^ CD4^−^) lymphocytes were not altered between 4 genotypes (Fig. 4a, b). The percentage of T_reg_ (CD45^+^SSC^low^CD3^+^CD8^−^CD4^+^CD25^+^Foxp3^+^) tended to be elevated in dystrophic animals (*mdx* and miR-146a^*−/−*^*mdx*) vs. their healthy counterparts (Fig. 4c). The lack of miR-146a did not change the level of T_reg_ cells between *mdx* and miR-146a^*−/−*^*mdx* (Fig. 4c). Accordingly, no changes were shown in the number of lymphocytes in the peripheral blood of mice of 4 genotypes (data not shown).

### miR-146a deficiency does not affect proliferation and differentiation of SCs

**Fig. 5:**
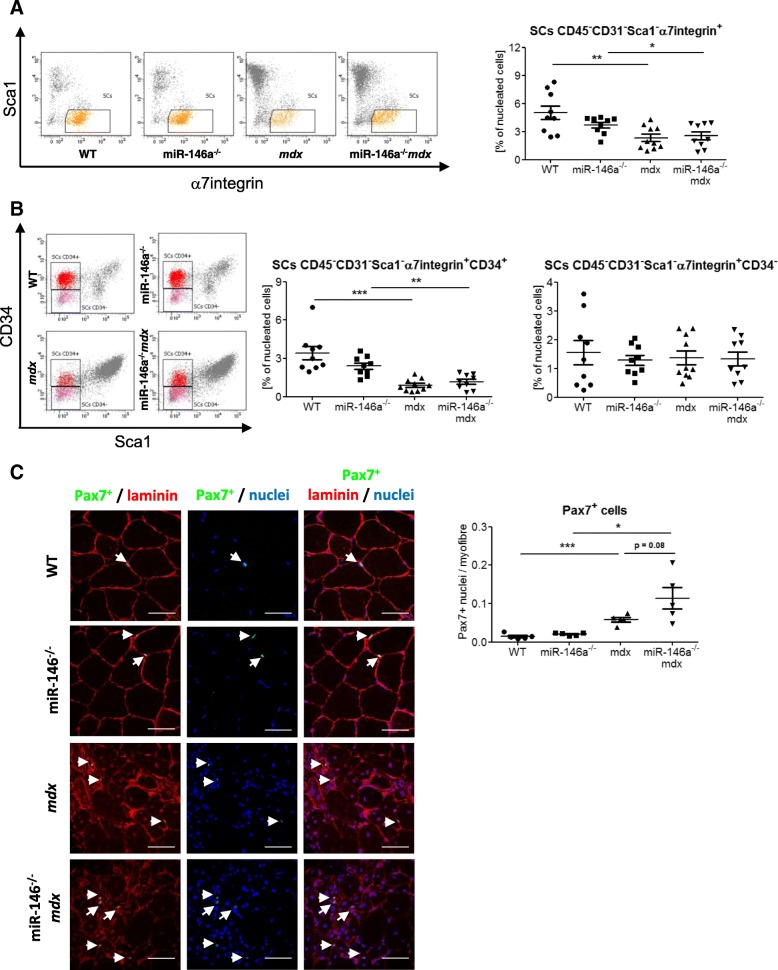
Number of SCs from WT, miR-146a^−/−^, *mdx*, and miR-146a^−/−^*mdx* hind limb muscles. **a** Percentage of SCs (CD45^−^CD31^−^Sca1^−^α7integrin^+^); flow cytometry. **b** Percentage of quiescent SCs (CD45^−^CD31^−^Sca1^−^α7integrin^+^CD34^+^) and activated SCs (CD45^−^CD31^−^Sca1^−^α7integrin^+^CD34^−^); flow cytometry. **c** Ratio of Pax7+ cells to myofibre, IHF staining; representative photos. Mean +/− SEM; *n* = 5–10; *− *p* ≤ 0.05; **− *p* ≤ 0.01; ***− *p* ≤ 0.001. Scale bars 50 μm

Since miR-146a was shown to affect proliferation of myoblasts [42], we analysed quantity, proliferation, and differentiation of SCs isolated from 4 genotypes. The percentage of SCs (CD45^−^CD31^−^Sca1^−^α7integrin^+^) among nucleated cells in the suspension of cells generated after enzymatic lysis of muscle tissue was reduced in *mdx* and miR-146a^*−/−*^*mdx* in comparison to WT and miR-146a^*−/−*^, respectively, though miR-146a deficiency in dystrophic animals did not change it further (Fig. 5a). Accordingly, the level of quiescent SCs (CD45^−^CD31^−^Sca1^−^α7integrin^+^CD34^+^) was decreased in dystrophic *mdx* and miR-146a^*−/−*^*mdx* mice, but the lack of miR-146a did not affect it additionally (Fig. 5b). The percentage of activated SCs (CD45^−^CD31^−^Sca1^−^α7integrin^+^CD34^−^) was not changed in mice of different genotypes (Fig. 5b).

Since flow cytometric results are calculated in relation to all nucleated cells, which are increased in dystrophic muscles due to heavy immune infiltration, assessment of the absolute number of SCs in muscles by IHF staining of Pax7 on muscle sections was additionally performed. The number of Pax7^+^ cells was calculated in relation to the total number of myofibres, as a more stable parameter among genotypes than the number of nuclei. The absolute number of SCs calculated by this method is increased in dystrophic muscles (Fig. 5c). Importantly, regardless of the method used, there is no effect of miR-146a deficiency on SCs count.

**Fig. 6:**
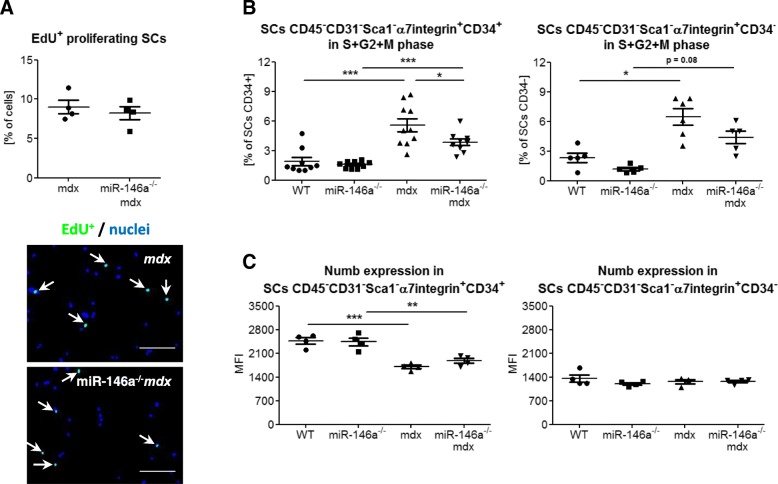
**a** Proliferation of SCs from WT, miR-146a^−/−^, *mdx*, and miR-146a^−/−^*mdx* hind limb muscles. Percentage of in vitro proliferating (EdU^+^) SCs (CD45^−^CD31^−^Sca1^−^α7integrin^+^); ICC-F staining; representative photos. **b** Percentage of proliferating SCs (CD45^−^CD31^−^Sca1^−^α7integrin^+^CD34^+^ and CD45^−^CD31^−^Sca1^−^α7integrin^+^CD34^−^); flow cytometry. **c** Numb expression in SCs (CD45^−^CD31^−^Sca1^−^α7integrin^+^CD34^+^ and CD45^−^CD31^−^Sca1^−^α7integrin^+^CD34^+^); flow cytometry. Mean +/− SEM; *n* = 4–10; *− *p* ≤ 0.05; **− *p* ≤ 0.01; ***− *p* ≤ 0.001. Scale bars 100 μm

FACS-sorted SCs (CD45^−^CD31^−^Sca1^−^α7integrin^+^) were cultured for 1 day in vitro and then proliferation was analysed by incorporation of EdU into DNA of cells remaining in S-phase (Fig. 6a). We did not observe differences between SCs of *mdx* and miR-146a^*−/−*^*mdx* (Fig. 6a). Proliferation was also analysed in CD45^−^CD31^−^Sca1^−^α7integrin^+^CD34^+^ and CD45^−^CD31^−^Sca1^−^α7integrin^+^CD34^−^ cells by flow cytometry assessment of cells in S + G2M phases basing on an increased level of Hoechst incorporation (Fig. 6b). The proliferation of cells from dystrophic muscles was increased and additionally, in the case of CD45^−^CD31^−^Sca1^−^α7integrin^+^CD34^+^ SCs, the lack of miR-146a reduced it in comparison to *mdx* animals. The level of Numb protein, the target of miR-146a [42], was also analysed in quiescent and activated SCs (Fig. 6c). Its decreased level was observed in CD45^−^CD31^−^Sca1^−^α7integrin^+^CD34^+^ isolated from dystrophic animals, whereas no differences were evoked by the additional lack of miR-146a in *mdx* animals (Fig. 6c). We found no differences in Numb expression in activated SCs (Fig. 6c).

**Fig. 7:**
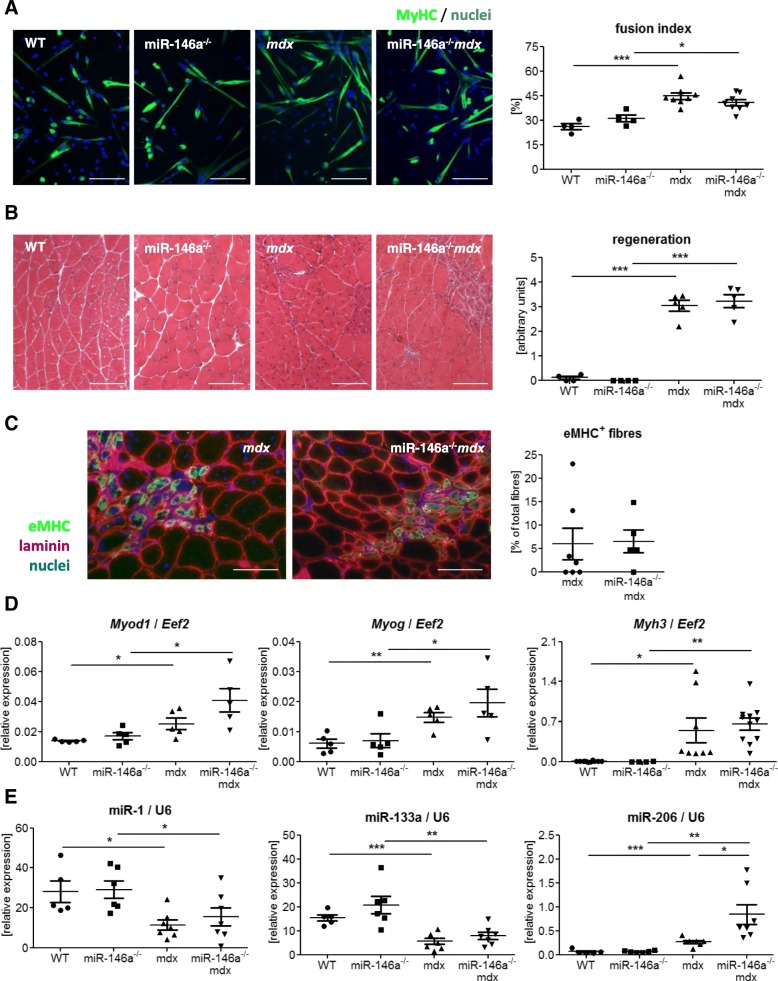
Differentiation of SCs and regeneration of GM muscles WT, miR-146a^−/−^, *mdx*, and miR-146a^−/−^*mdx* mice. **a** Fusion index of in vitro differentiated SCs (CD45^−^CD31^−^Sca1^−^α7integrin^+^); ICC-F; representative photos. **b** Semi-quantitative analysis of centrally nucleated myofibres in GM; HE staining; representative photos. **c** Analysis of eMHC^+^ myofibres in GM; IHF staining; representative photos. **d** *Myod1*, *Myog*, *Myh3*, **e** miR-1, miR-133a, miR-206 level in GM; qRT-PCR. Mean +/− SEM; *n* = 4–11; *− *p* ≤ 0.05; **− *p* ≤ 0.01; ***− *p* ≤ 0.001. Scale bars 100 μm

To analyse the differentiation potential of SCs, CD45^−^CD31^−^Sca1^−^α7integrin^+^ cells were FACS-sorted and subjected to in vitro culture in DMEM medium supplemented with 2% horse serum. SCs from *mdx* and miR-146a^*−/−*^*mdx* formed multinucleated myotubes more frequently than the appropriate control animals, but no differences were visible between both dystrophic genotypes (Fig. 7a). Moreover, neither histological examination of regenerating myofibres (with centrally located nuclei, Fig. 7b) nor IHF staining of maturating myofibres (expressing eMHC, Fig. 7c), revealed differences between *mdx* and miR-146a^*−/−*^*mdx* muscles. In a qRT-PCR analysis of markers of differentiation, increased expression of *Myod1*, *Myog*, and *Myh3* in *mdx* vs. WT and miR-146a^*−/−*^*mdx* vs. miR-146a^*−/−*^ was observed, with no effect of miR-146a deficiency (Fig. 7d). Expression of miR-1 and miR-133a was downregulated in dystrophic animals, whereas the opposite effect was found in the case of miR-206 (Fig. 7e). Additionally, miR-206 was increased in miR-146a^*−/−*^*mdx* in comparison to *mdx* animals (Fig. 7e).

miR-206 is not only involved in muscle development, but it may also play a role in the regulation of the angiogenesis process, mostly through the repression of proangiogenic VEGF [53–55]. Accordingly, downregulation of miR-206 in *mdx* mice was shown to significantly increase both the VEGF transcript and protein level [56]. Furthermore, in the in silico studies, *Vegfa* is shown as one of the predicted targets of miR-206 (miR-206-3p strain). Thus, although we did not observe changes in *Vegfa* on mRNA level in gastrocnemius muscle (Additional file 1: Figure S1A), a significant decrease of VEGF protein was evident in *mdx* vs. WT counterparts and was further diminished in *mdx* mice additionally lacking miR-146a (Additional file 1: Figure S1B). Interestingly, the potential impact of miR-146a on the regulation of another pro-angiogenic factor, namely stromal cell-derived factor-1α (SDF-1α, *Cxcl12* gene), was revealed, as the diminished level of *Cxcl12* in miR-146a^−/−^ vs. WT animals was noted (Additional file 1: Figure S1C).

### miR-146a deficiency upregulates *Tgfb1* expression but does not affect collagen deposition in dystrophic muscles

**Fig. 8:**
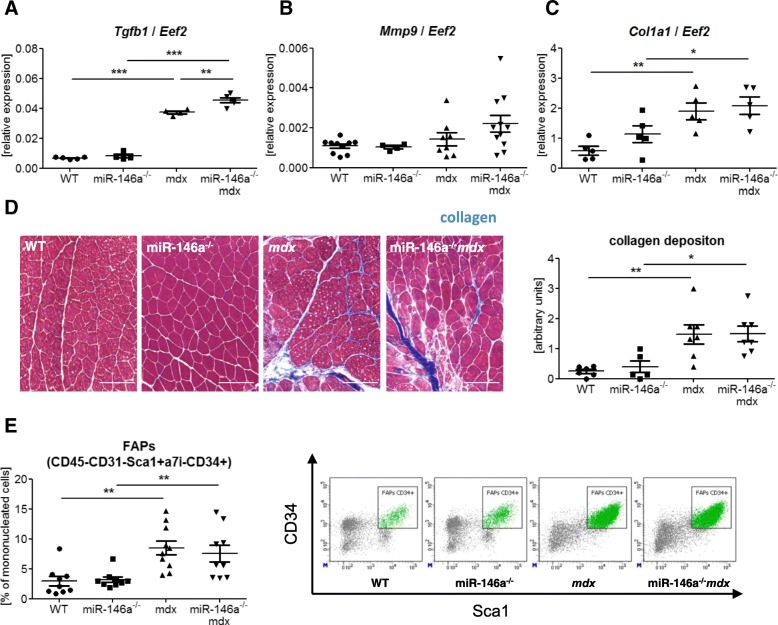
Fibrosis in WT, miR-146a^−/−^, *mdx*, and miR-146a^−/−^*mdx* hind limb muscles. **a** *Tgfb1*, **b** *Mmp-9*, and **c** *Col1a1* level in GM; qRT-PCR. **d** Semi-quantitative analysis of collagen deposition in GM; trichome staining; representative photos. **e** Percentage of FAPs; (CD45^−^CD31^−^Sca1^+^α7integrin^−^CD34^+^); flow cytometry. Mean +/− SEM; *n* = 5–11; *− *p* ≤ 0.05; **− *p* ≤ 0.01; ***− *p* ≤ 0.001. Scale bars 100 μm

miR-146a was shown to act as a negative regulator of TGF-β signalling pathway affecting the fibrosis process [39, 40, 57]. Accordingly, we have found increased *Tgfb1* mRNA level in *mdx* vs. WT mice which was further accelerated in *mdx* mice additionally lacking miR-146a (Fig. 8a), suggesting that the deficiency of miR-146a could increase fibrosis also in our model. Nonetheless, no difference in mRNA level of another pro-fibrotic factor, *Mmp9*, that was shown to be inhibited by the miR-146a in human cardiac cells [58], in miR146a^−/−^*mdx* mice in comparison to *mdx* animals was visible (Fig. 8b). Additionally, although the expression of *Col1a1* (Fig. 8c) and collagen deposition assessed by Masson’s trichrome staining followed by the arbitrary analysis (Fig. 8d) were increased in dystrophic mice, we did not observe further induction in muscles additionally lacking miR-146a. Finally, the level of FAPs (CD45^−^CD31^−^Sca1^+^α7integrin^−^CD34^+^) (Fig. 8e) was upregulated by dystrophin deficiency but was not affected by miR-146a absence.

### miR-146a deficiency does not aggravate dystrophy progression in 24-week-old animals

Moreover, we have performed additional analysis in the older, 24-week-old mice (Additional file 2: Figure S2). However, our results do not show further aggravation of the dystrophic phenotype. Although LDH (Additional file 2: Figure S2 A) and CK (Additional file 2: Figure S2 B) activity are still potently elevated in *mdx* vs. WT counterparts, additional lack of miR-146a does not further accelerate the level of muscle damage markers in serum. Obtained results were strengthened by the analysis of inflammation extent and regeneration in the gastrocnemius muscle (based on the HE staining), which consistently show no effect of the lack of miR-146a on typical aspects of DMD pathology (Additional file 2: Figure S2 C, D). Hence, our results indicate that the lack of miR-146a does not affect the progression of DMD with age in *mdx* mice.

## Discussion

DMD is one of the most extensively described, inherited disorders of the childhood. Despite its relatively high frequency of occurrence and well-known both genetic and molecular background, the disease is incurable and life quality of DMD patients is significantly compromised particularly in the last stages. Although the gene and cell therapy were hoped to provide the ultimate cure for the disease, technical obstacles and safety problems have made them so far not effective enough [11, 25, 59]. Therefore, therapeutic strategies that are at present examined are focused on ameliorating the destructive effects of the disorder, not the dystrophin deficiency itself, and as such, they require a long-term application [11, 25, 59]. For instance, a current gold standard for treatment of DMD are corticosteroids, which due to their anti-inflammatory effects provide stabilisation of muscle strength and function, promote independent ambulation, and delay the onset of scoliosis and cardiomyopathy [25, 59]. However, they also result in weight gain, gastrointestinal symptoms, and metabolic disorders as well as osteoporosis, and thus chronic corticosteroids application is not well tolerated by some patients [25, 59]. Therefore, compounds that can potentially diminish progressive muscle damage by targeting inflammatory reaction, innate immunological response, muscle regeneration, and fibrosis are constantly analysed in animal studies and clinical trials, to find a better and more effective cure for the disease [11, 25]. In this context, profound and comprehensive knowledge of the mechanisms regulating the pathogenesis of DMD may help in the successful search for factors modulating them. Since miR-146a is a factor that was previously shown to diminish inflammation in different tissues [26–32, 35–38, 40], inhibit muscle fibrosis [39, 40], and induce proliferation of myoblasts [42], we have examined its role in the disease progression in the murine model of DMD—*mdx* mice.

The effects of miR-146a in skeletal muscles have been found mostly in the context of its anti-inflammatory function so far. It is elevated in myositis muscles [32] and upregulated in skeletal muscles in response to lipopolysaccharide [60] or TNF-like weak inducer of apoptosis (TWEAK) induction [61]. We have recently demonstrated that miR-146a is raised in dystrophic muscles [9], whereas others showed that it is decreased by steroid treatment [62]. In the current model, this result was also confirmed—we observed increased miR-146a expression in *mdx* mice vs. WT. Although miR-146a was also described to reduce the translation of dystrophin [63], we did not observe the induction of dystrophin in mice lacking miR-146a. In line with that, no differences in the level of muscle degeneration (muscle necrosis and plasma activity of LDH) were found in miR-146a-deficient animals, namely miR-146a^−/−^ or miR-146a^−/−^*mdx* vs. WT or *mdx*, respectively. Only a muscle-specific marker of muscle damage, CK, was increased in 12-week-old miR-146a^−/−^*mdx* vs. dystrophic animals, but this difference disappeared in 24-week-old animals. Hence, miR-146a can partially ameliorate disease severity in the younger *mdx* mice, when the dystrophic phenotype is stronger. However, it does not appear to aggravate disease progression in older *mdx* mice, known to demonstrate stabilisation of muscle pathology.

Since in DMD patients the induction of innate immunological response was shown to occur soon after the birth, before the onset of muscle-related clinical symptoms [11], we decided to analyse the major cellular components taking part in this process. Accordingly, though apart from CK no differences in muscle degeneration in miR-146a-deficient mice were observed, increased expression of proinflammatory cytokines (*Il1b, Ccl2, Tnf*) in muscles lacking miR-146a was noted. Moreover, in GM of miR-146a^−/−^*mdx*, we observed a tendency to an increased inflammatory reaction, based on semi-quantitative analysis of HE staining. However, macrophages, which are the major population infiltrating dystrophic muscles [15], remained unchanged upon additional deletion of miR-146a in *mdx* mice. Similarly, monocytes, as well as M1-like and M2-like macrophage subtypes, were increased in dystrophic muscles, but no differences were detected between miR-146a^−/−^*mdx* and *mdx* animals. miR-146a was previously demonstrated to inhibit the activity of the NF-κB pathway [26–28] and production of proinflammatory cytokines [30–32], among others prominent chemoattractant for monocytes/macrophages—CCL2 (C-C motif chemokine ligand 2) [52]. Consequently, increased monocyte and macrophage number were detected in the spleen of 12-month-old mice lacking miR-146a [30] and in a rat model of polymyositis with decreased miR-146a level [35]. In our model of muscular dystrophy with miR-146a deficiency, the lack of similar differences in skeletal muscle may result from the higher miR-206 expression that was detected in miR-146a^−/−^*mdx* mice in comparison to *mdx* animals, as this microRNA was shown to directly diminish CCL2 expression [64, 65]. Previously, the effect miR-146a was shown to suppress mainly the activity of NK cells [33, 34], the function of T_reg_ [36, 37], and the resolution of T cell response [29, 38]. However, similarly to macrophages, no differences were found in number of T (CD45^+^SSC^lo^CD3^+^NK1.1^−^), T_h_ (CD45^+^SSC^lo^CD3^+^CD4^+^CD8^−^), T_c_ (CD45^+^SSC^lo^CD3^+^CD4^−^CD8^+^), and T_reg_ (CD45^+^SSC^lo^CD3^+^CD4^+^CD8^−^Foxp3^+^CD25^+^) lymphocytes and NK cells (CD45^+^SSC^lo^CD3^−^NK1.1^+^) infiltrating the skeletal muscle of mice of different miR-146a genotype.

Little is known about the function of miR-146a in skeletal SCs and myoblast. So far, it was demonstrated that miR-146a is connected to the increased proliferation and decreased differentiation, but the studies were done in C2C12 myoblasts cell lines [41, 42]. In the current research, we have therefore investigated the effect of miR-146a deficiency on primary muscle SCs. Although we have observed similarly disturbed SCs differentiation in *mdx* mice as in previous study [9], we did not find any influence of additional lack of miR-146a on number of SCs (CD45^−^CD31^−^Sca1^−^α7integrin^+^), quiescent SCs (CD45^−^CD31^−^Sca1^−^α7integrin^+^CD34^+^), and activated SCs (CD45^−^CD31^−^Sca1^−^α7integrin^+^CD34^−^). To verify these results, we performed also the analysis of Pax7^+^ cells in muscle sections confirming the lack of effect of miR-146a on SCs quantity. Of note, contrary to SCs’ number calculated in relation to all nucleated cells in flow cytometric analysis, Pax7^+^ staining revealed that the number of SCs counted per myofibre is increased in *mdx* animals. It should be, however, remembered that calculation of SCs as a percentage of nucleated cells in dystrophic muscles, strongly infiltrated by immune cells, results in a reduction of SCs’ percentage that can create a discrepancy in the interpretation of the effect of DMD on satellite cells [20, 66, 67].

Though the proliferation of miR-146a^−/−^*mdx* CD34^+^ SCs was decreased, it was not confirmed by in vitro incorporation of EdU compound or proliferation of CD34^−^ SCs. In line with that, Numb expression, which was previously shown to be targeted by miR-146a [42], was not changed in miR-146a^−/−^ and miR-146a^−/−^*mdx* vs. WT or *mdx*, respectively. Concomitantly, there were neither differences in ex vivo differentiation potential of FACS-sorted SCs lacking miR-146a nor in the rate of regeneration in GM of miR-146a-deficient mice. The expression of major MRFs (MyoD, myogenin), proteins specific for regenerating fibres (eMHC), and myomirs (miR-1, miR-133a) was also not affected, whereas miR-206 was upregulated in miR-146a^−/−^*mdx* vs. *mdx*. We did not, however, observe the beneficial effects of increased miR-206 expression on muscle regeneration that were previously demonstrated in dystrophic muscles [48, 68].

Interestingly, a recent paper by Bulaklak et al. suggested also another role for miR-206 in dystrophic muscles [56]. AAV-mediated miR-206 inhibition was able to attenuate dystrophic phenotype in *mdx* mice as improved motor deficits and running capacities were observed. Importantly, this effect was also associated with the induction of angiogenic response by increased *Vegfa* mRNA level and improved vascularisation. In our hands, increased expression of miR-206 in miR-146a^−/−^*mdx* mice correlated with a diminished protein level of VEGF in muscles isolated from mice lacking dystrophin and miR-146a. This could be, at least partially, explained by the increased miR-206 expression.

Recent findings revealed that the impairment in angiogenic response and alteration in angiogenic mediators might highly contribute to DMD pathology (reviewed in [69]). Therefore, the modulation of angiogenesis process has been already considered as a therapeutic strategy to ameliorate DMD progression. Interestingly, some studies already revealed the involvement of miR-146a in the blood vessel formation [70, 71]. The changes of *Vegfa* and *Cxcl12* expression noted in our studies warrants further investigations on the role of miR-146a in angiogenesis.

As in many chronic inflammatory disorders, also in DMD, increased level of TGF-β is observed, associated with the fibrotic replacement of muscle tissue [11]. Importantly, miR-146a was shown to act as a negative regulator of TGF-β signalling pathway and to inhibit fibrous scar formation in skeletal and cardiac muscle [39, 40]. In accordance, we observed that *Tgfb1* expression was increased in miR-146a deficient *mdx* mice muscles. We have also noted an augmented collagen deposition, number of FAPs, and expression of collagen 1α in both *mdx* and miR-146a^−/−^*mdx* animals in comparison to WT and miR-146a^−/−^ mice, respectively. Noteworthy, the above parameters were unaffected by the lack of miR-146a itself when compared to the WT counterparts, as well as in *mdx* mice devoid of miR-146a, undermining the impact of the global lack of miR-146a on muscle fibrosis in 12-week-old dystrophic mice.

Moreover, to investigate the effects of miR-146a deficiency in older animals, we have performed additional analysis in the 24-week-old mice. However, further aggravation of the muscle degeneration, inflammation, and regeneration was not observed. Hence, our results indicate that the lack of miR-146a does not affect the progression of DMD with age. However, we have to be aware that the *mdx* mice only partially reflect the human DMD and present milder muscle phenotypes of inflammation and fibrosis comparing to human patients. The mice display minimally shortened lifespan; the muscle damage and regeneration is evident in young animals, but after 3 months of age, it is stabilised and does not strongly progress [72–74]. Therefore, one may suggest that the role of miR-146a can be better visible in other DMD animal models, in which typical symptoms of the disease related to inflammation and fibrosis are more severe [72–74].

## Conclusions

miR-146a is increased in dystrophic muscles, and its lack in *mdx* mice is associated with the aggravation of some of the markers of muscle damage and inflammation. Additionally, the deficiency of miR-146a increases *Tgfb1* expression while decreases *Vegfa* in dystrophic muscles. Nevertheless, knockout of miR-146a does not evoke significant changes in skeletal muscle degeneration and regeneration in *mdx* model.

## Additional files

Additional file 1: Figure S1 Angiogenic gene expression in WT, miR-146a^−/−^, *mdx* and miR-146a^−/−^*mdx* mice. (A) *Vegfa* mRNA level in GM; qRT-PCR, (B) VEGF protein level; Luminex analysis (C) *Cxcl12* mRNA level in GM; Mean +/− SEM; *n* = 4–6. (PDF 44 kb)

Additional file 2: Figure S2 The analysis of degeneration, inflammation and regeneration of 24-week-old WT, miR-146a^−/−^, *mdx* and miR-146a^−/−^*mdx* mice. The activity of (A) LDH and (B) CK in plasma; activity test. Semi-quantitative analysis of (C) inflammation and (D) centrally nucleated myofibres in GM; HE staining. Mean +/− SEM; *n* = 6–12; * - *p* ≤ 0.05; ** - *p* ≤ 0.01; *** - *p* ≤ 0.001. Scale bars: 100 μm. (PDF 69 kb)

## Data Availability

The datasets used and/or analysed during the current study are available from the corresponding author on reasonable request.

## Abbreviations

CCL2: C-C motif chemokine ligand 2
CK: Creatine kinase
DMD: Duchenne muscular dystrophy
ECM: Extracellular matrix
eMHC: Embryonic myosin, Myh3
FAPs: Fibro-adipogenic progenitors
GM: Gastrocnemius muscle
HE: Haematoxylin and eosin
ICC-F: Immunocytochemical fluorescent staining
IHF: Immunohistofluorescent
IFNγ: Interferon-γ
IRAK1: Interleukin-1 receptor-associated kinase 1
LDH: Lactate dehydrogenase
MRFs: Muscle regulatory factors
MyHC: Myosin-heavy chain
SCs: Satellite cells
SDF-1α: Stromal-derived factor-1α
TGF-β: Transforming growth factor-β
TNFα: Tumour necrosis factor-α
TRAF6: TNF receptor-associated factor 6
TWEAK: TNF-like weak inducer of apoptosis
