# Supplementary figures and images for: miR-146a deficiency does not aggravate muscular dystrophy in mdx mice
Source: Skelet Muscle. 2019 Aug 14;9:22. doi: 10.1186/s13395-019-0207-0 (PMC6693262; doi:10.1186/s13395-019-0207-0)

Additional file 1: Figure S1

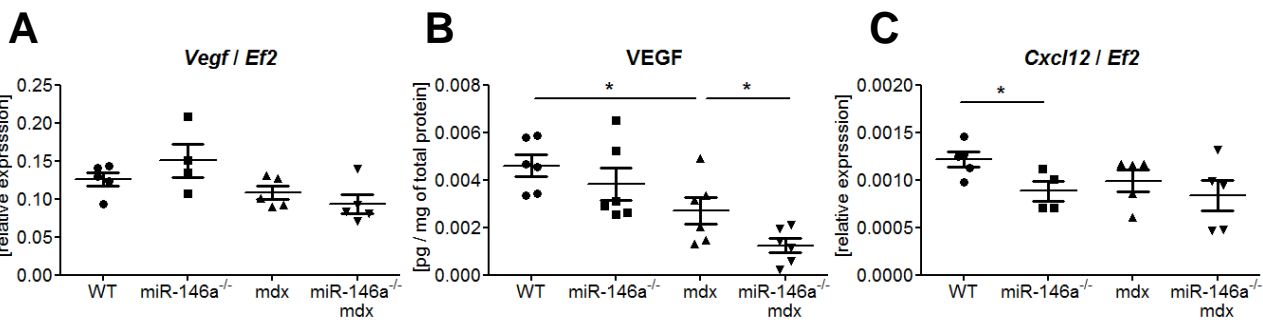

Supplement: Supplementary file 1 — Figure S1 Angiogenic gene expression in WT, miR-146a−/−, mdx and miR-146a−/−mdx mice. (A) Vegfa mRNA level in GM; qRT-PCR, (B) VEGF protein level; Luminex analysis (C) Cxcl12 mRNA level in GM; Mean +/− SEM; n = 4–6. (PDF 44 kb) [file 13395_2019_207_MOESM1_ESM.pdf]

# Additional file 2: Figure S2

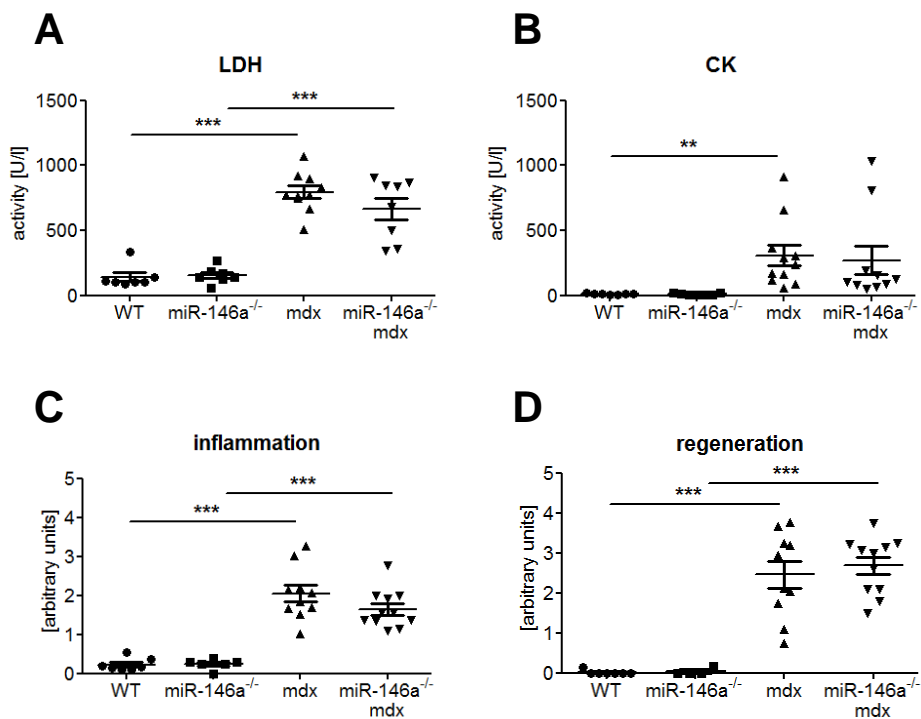

Supplement: Supplementary file 2 — Figure S2 The analysis of degeneration, inflammation and regeneration of 24-week-old WT, miR-146a−/−, mdx and miR-146a−/−mdx mice. The activity of (A) LDH and (B) CK in plasma; activity test. Semi-quantitative analysis of (C) inflammation and (D) centrally nucleated myofibres in GM; HE staining. Mean +/− SEM; n = 6–12; * - p ≤ 0.05; ** - p ≤ 0.01; *** - p ≤ 0.001. Scale bars: 100 μm. (PDF 69 kb) [file 13395_2019_207_MOESM2_ESM.pdf]
